# Rapid in-solution preparation of somatic and meiotic plant cell nuclei for high-quality 3D immunoFISH and immunoFISH-GISH

**DOI:** 10.1186/s13007-023-01061-7

**Published:** 2023-08-08

**Authors:** Diána Makai, Edit Mihók, Dávid Polgári, András Cseh, Andrea Lenykó-Thegze, Adél Sepsi, László Sági

**Affiliations:** 1 Centre for Agricultural Research, Eötvös Loránd Research Network, Martonvásár, 2462 Hungary; 2 Doctoral School of Plant Sciences, Hungarian University of Agriculture and Life Sciences, Gödöllő, 2100 Hungary; 3 Institute of Genetics and Biotechnology, Hungarian University of Agriculture and Life Sciences, Gödöllő, 2100 Hungary; 4 Doctoral School of Biology, Eötvös Loránd University, Budapest, 1117 Hungary; 5 Agribiotechnology and Precision Breeding for Food Security National Laboratory, Plant Biotechnology Section, Centre for Agricultural Research, Martonvásár, 2462 Hungary

**Keywords:** Barley, Cereal, Immunolabelling, In situ hybridisation, Interphase nuclei, Wheat

## Abstract

**Background:**

Though multicolour labelling methods allow the routine detection of a wide range of fluorescent (immuno)probe types in molecular cytogenetics, combined applications for the simultaneous in situ detection of proteins and nucleic acids are still sporadic in plant cell biology. A major bottleneck has been the availability of high-quality plant nuclei with a balance between preservation of 3D ultrastructure and maintaining immunoreactivity.

The aim of this study was to develop a quick and reliable procedure to prepare plant nuclei suitable for various combinations of immunolabelling and fluorescence in situ hybridisation methods (immunoFISH-GISH).

**Results:**

The mechanical removal of the cell wall and cytoplasm, instead of enzymatic degradation, resulted in a gentle, yet effective, cell permeabilisation. Rather than manually releasing the nuclei from the fixed tissues, the procedure involves in-solution cell handling throughout the fixation and the preparation steps as ended with pipetting the pure nuclei suspension onto microscope slides. The optimisation of several critical steps is described in detail. Finally, the procedure is shown to be compatible with immunolabelling, FISH and GISH as well as their simultaneous combinations.

**Conclusion:**

A simple plant cell nuclei preparation procedure was developed for combined immunolabelling-in situ hybridisation methods. The main and critical elements of the procedure are: a short period of fixation, incorporation of detergents to facilitate the fixation of tissues and the penetration of probes, tissue grinding to eliminate unwanted cell components, and an optimal buffer to handle nuclei. The procedure is time efficient and is easily transferable without prior expertise.

**Supplementary Information:**

The online version contains supplementary material available at 10.1186/s13007-023-01061-7.

## Background

Microscopic imaging of the chromatin in the plant cell has greatly contributed to our understanding of its dynamic organisation and function in fundamental cellular processes such as transcription, DNA repair and recombination. Tracing the chromatin within the plant nucleus has uncovered the non-random organisation of chromosomes into distinct nuclear territories [1], where chromatin function is influenced by the dynamics and structural interactions between these territories.

The compartmentalisation of individual genomic sequences or the characteristic shaping of chromosome regions through different stages of the plant cell cycle (telomer/leptotene bouquet [2], Rabl configuration [3]), the spatial distribution of genes and transposable elements [4], and specific epigenetic states (DNA methylation, histone modifications) are all indispensable factors for the chromatin to express its complex functions. It is therefore essential that a complementary array of methods is available for the detection and dynamic monitoring of these factors [5].

Immunodetection and fluorescence in situ hybridisation (FISH) methods, which make use of labelled antibodies and nucleic acid probes, respectively, are powerful tools to study chromatin organisation and estimate the dynamics of specific genomic regions. Immunolabelling can primarily detect nuclear proteins and their variants while FISH provides a clear insight into the physical position of genomic loci or chromosome segments relative to the cell surface. Additionally, FISH reveals the average proximity between two or more genomic loci situated within the nuclear space in multiple types of individual cells and over various developmental stages. FISH is therefore a powerful means for testing experimentally the predictions of structural models on the global nuclear architecture [6, 7]. FISH and its whole genome flavour (GISH) have become standard tools for the physical mapping of repetitive DNA sequences, multi-copy and even single-copy genes in plant chromosomes, and for the characterisation of full chromosome complements or translocated segments, respectively, in artificial hybrid as well as natural (paleo)allopolyploid genomes [8]. Based on these properties, chromosome in situ hybridisation has been applied in wide-ranging fields of plant biology research from chromosome evolution [9, 10] to genome diversity and phylogeny [11], and even to genome sequencing [12].

Though recent advances in high- and super-resolution microscopy allow imaging and resolving nuclear arrangements in multiple colours and three dimensions (3D) at the nanoscale, chromatin segments together with other functional proteins require preparative and labelling methods to achieve high-resolution fluorescence signals. Imaging the plant nucleus is further encumbered by the presence of the cell wall, which blocks the access of protein antibodies and/or labelled probes within the cell. The major challenge in combining immunodetection with FISH lies in the apparently contradictory requirement to preserve the antigen epitope(s) detected by the antibody as well as the 3D structure of the nucleus but also to allow the penetration of the DNA probe to detect gene loci or chromosomal sub-regions [13].

The standard procedures of plant nuclei preparations for immunohistochemistry and molecular cytology follow (a) a non-denaturing tissue fixation to preserve nuclear proteins and general ultrastructure, (b) an enzymatic treatment to remove the plant cell wall/cytoplasm, and (c) manual tissue maceration to release cells on the surface of microscope slides [14–16].

Formaldehyde treatment is a widespread fixation method to preserve cellular proteins, chromatin structure, and nuclear integrity/morphology within living tissues [17, 18]. Depending on the aim of analysis, fixative concentration and treatment duration need to be adapted for each plant species or even genotype. The effectiveness of cell wall degradation depends on enzyme quality, which can vary among the manufacturers (or even batches of the same manufacturer). In addition, enzyme activities will significantly decrease or get lost with time, especially when reused and undergone several freeze-thaw cycles. Finally, concentrations, dilution buffers, pH, temperatures, and duration times need to be optimised for the enzyme vendor and genotypes, representing a further lack of control in the methodology. An additional complication in the preparation of nuclei for microscopic observation is the great deal of skill and training required for the manual release of cells (by needles, brass rods, or tweezers) from intact tissues onto the microscope slides, which are indispensable to produce high-quality specimens.

To address the above limitations, we developed a simple nuclei preparation procedure where fixation is adjusted to preserve the 3D ultrastructure, while cell wall/cytoplasm are removed mechanically, without the use of dedicated enzymes. To allow the transferability of the present procedure, manual maceration of the intact tissues was substituted with tissue grinding. This procedure was tested with interphase/prophase nuclei from root tips and male meiocytes of small grain cereals (wheat, barley, and rye) to obtain specimens suitable for both immunolabelling and in situ hybridisation methods as well as for their combinations, but it can be applied to different plant species and cell types.

## Results

**Fig. 1:**
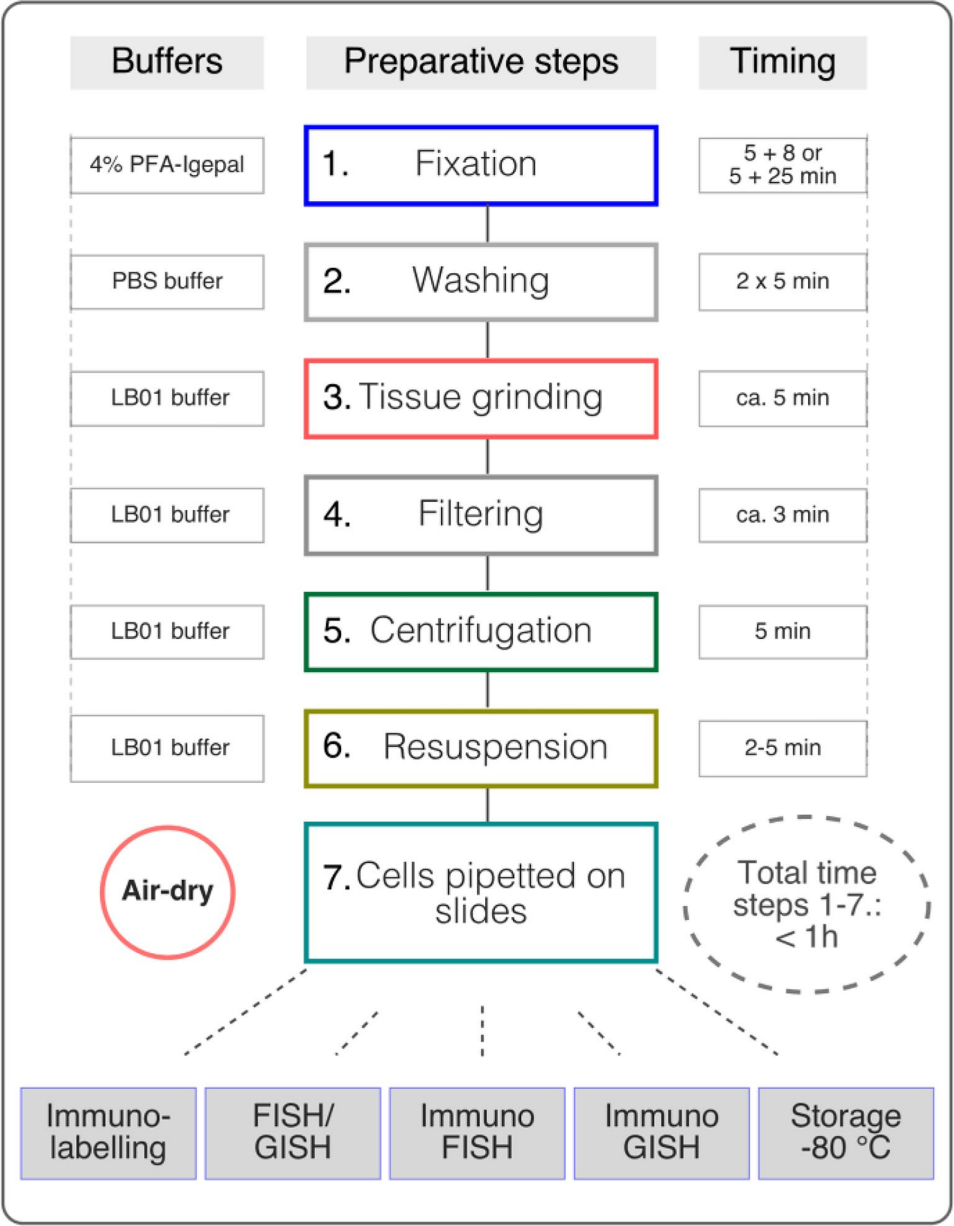
Flowchart of the nuclei preparation procedure suitable for protein visualisation by immunolabelling and localisation of nucleic acid sequences by in situ hybridisation. The main steps of the procedure are numbered in the middle in coloured boxes. Buffers and the respective timing are indicated on the left and right side, respectively. Possible downstream applications are indicated in grey boxes in the bottom

The procedure was devised to ensure a practical in-solution tissue treatment (Fig. 1: steps 1–7) that preserves antigen epitopes, chromatin structure, and nuclear integrity via a concomitant removal of extranuclear cell components (cell wall and cytoplasm) by mechanical force (grinding and/or maceration) instead of applying cell wall degrading enzymes.

### Short fixation for high-quality nuclei preparations

This method consists of a short treatment in paraformaldehyde (PFA), an additive and non-coagulant fixative, followed by thorough (2 × 5 min) washing (Fig. 1: steps 1–2). To efficiently remove the cytoplasm by mechanical force alone, fixation times (1 h in 4% PFA) used in our standard procedure had to be reduced.

We first tested whether detergent added to the non-denaturing fixative influenced the quality of nuclei preparation. To this end, wheat and barley root tips were fixed for approx. 15 min in 4% PFA with or without adding 0.5% Igepal, a non-ionic and non-denaturing detergent, to the fixative solution. Penetration and fixation were aided by vacuum infiltration for 5 min in all treatments. Short fixation without detergent resulted in a poor nuclear morphology for both wheat and barley with considerably damaged cells (Additional file 1: Fig. S1A, left panel). The inclusion of the detergent (0.5% Igepal) readily improved nuclear morphology and reduced the number of damaged cells, although did not diminish it entirely (Additional file 1: Fig. S1A, right panel). The 3D structure of the nuclei and the integrity of the chromatin was significantly improved as well (Additional file 1: Fig. S1B).

The proportion of the damaged cells in wheat fixed without the addition of a detergent reached 80% while 0.5% Igepal significantly reduced the proportion of damaged cells to 17% of the total cell population (Additional file 1: Fig. S1C left: binomial test, p <0.001, n =122). Likewise, 57% of barley nuclei fixed without a detergent were damaged whereas the addition of 0.5% of Igepal significantly reduced the proportion of damaged cells to 18% (Additional file 1: Fig. S1C right, binomial test, p <0.001, n =159).

Having determined the key role of detergents during fixation, we searched for the optimal duration of fixation to further increase 3D structural preservation and potentially reduce the quantity of damaged nuclei or debris. Three different fixation times were applied to wheat and barley root tips (Additional file 1: Fig. S2A and B, resp.): 60 (5 min vacuum + 55) min, 30 (5 + 25) min, and 13 (5 + 8) min. We looked for the most favourable 3D structure with an adequate nuclear permeability as achieved via the subsequent mechanical removal of cell wall and cytoplasm. The 60 min fixation (Additional file 1: Fig. S2A, B, first columns) resulted in an excellent 3D structure, but the mechanical forces applied were not effective in removing the cytoplasm. This compromised permeability of the nucleus was confirmed by immunoFISH (Additional file 1: Fig. S2A, B, first columns, bottom images), which showed high background staining and low signal intensity both for the antibody and the FISH probes. Fixations of 30 min and 13 min both preserved the nuclear structure and also allowed a good antibody and probe penetration (Additional file 1: Fig. S2A, B, second and third columns, and Fig. 3, Fig. 5). The 30 min of fixation had the advantage of producing a lower proportion of damaged cells and debris, especially in wheat (Additional file 1: Fig. S2A), and it was therefore considered as an optimal fixation time for the genotypes used in the present study.

### Tissue homogenisation and sample preparation

Tissue homogenisation in a dedicated grinding set (Fig. 1: step 3, see the M&M section) offers the advantage to process large amounts of plant material in a short period of time, to reduce the need for rigorous manipulations, and to improve cell permeabilisation. The introduction of tissue grinding makes the method easily transferable and reproducible, with providing good quality nuclei preparations. Cells, however, are prone to form aggregates in suspensions, which prevents successful cytoplasm removal, effective cell filtration and causes high background staining in molecular cytology methods. To reduce cell aggregation, tissue grinding was performed in different solutions to identify the most suitable composition for cell separation.

Three solutions, PEM (or BRB80) buffer [19], extraction solution (0.2 M mannitol, 0.15 M glucose, 2 mM CaCl_2_), and LB01 buffer [20] (see the “Materials and methods” section) were tested for this purpose. Tissue grinding in the PEM buffer and the extraction solution resulted in large cell aggregates and hardly any individual nuclei in barley (Additional file 1: Fig. S3). Samples in LB01, however, contained a high proportion of individual nuclei indicating an adequate cell wall/cytoplasm elimination (Additional file 1: Fig. S3).

The homogenised cells were double-filtered through a 70-µm and a 40-µm strainer (Fig. 1: step 4), then pelleted by centrifugation for 5 min at 2000×*g* (Fig. 1: step 5). The pelleted nuclei were resuspended in LB01 buffer (Fig. 1: step 6), pipetted on the surface of an adhesion slide and air dried (Fig. 1: step 7). The slides were then directly processed or stored at − 80 °C for several months.

### Optimised steps during in situ hybridisation

**Fig. 2:**
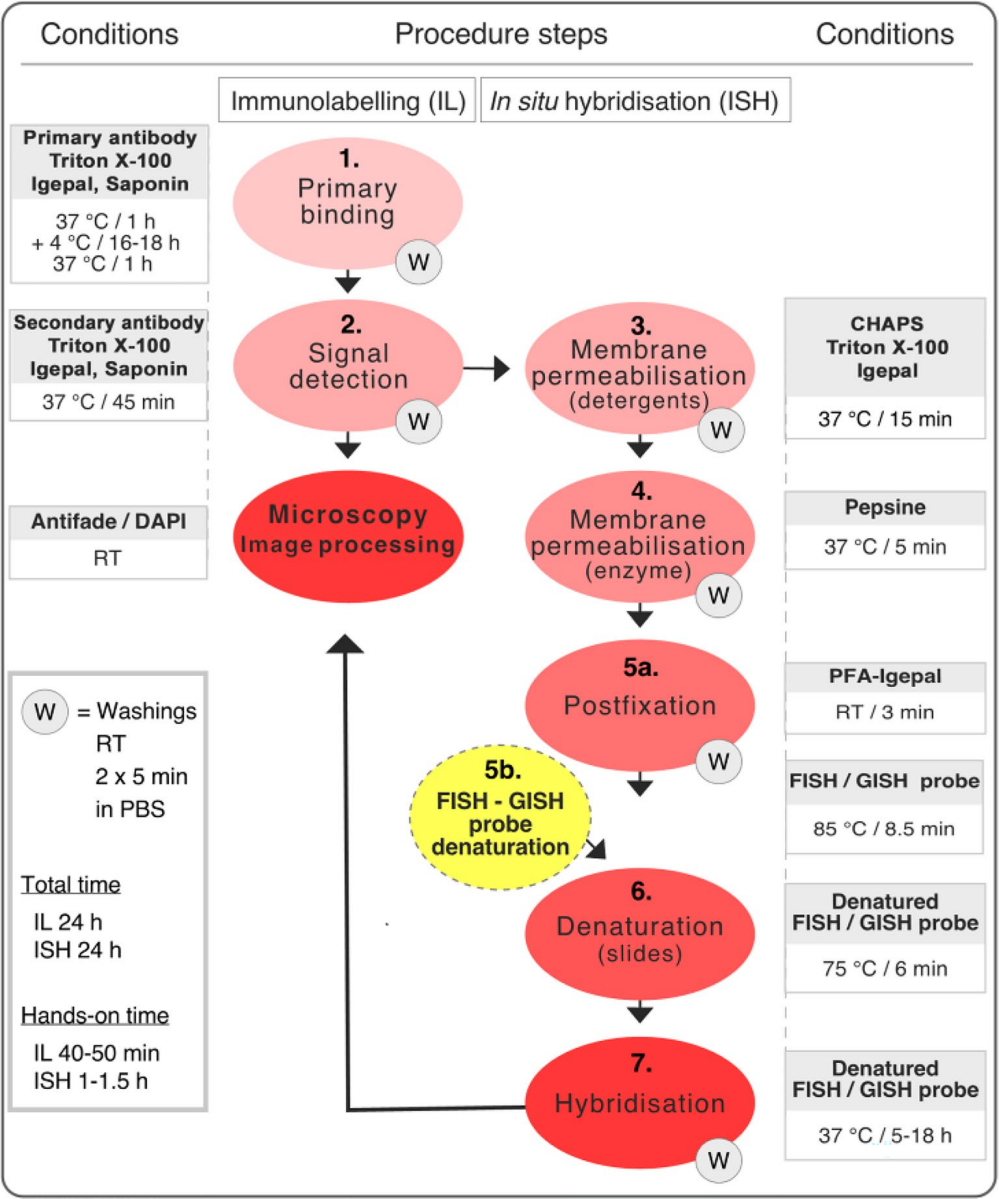
Flowchart of the immunolabelling and immunoFISH-GISH methodology (modified from [21]). The main steps are numbered in the middle in coloured boxes. The treatment conditions for immunolabelling and FISH-GISH are indicated on the left and right side, respectively

Performing immunolabelling with the blocking solution containing detergents (Fig. 2: step 1) significantly improved the efficiency of the subsequent in situ hybridisation procedure. However, the detection of specific DNA sequences by FISH or whole genomes by GISH required further permeabilisation.

Therefore, pre-FISH/GISH permeabilisation of interphase/prophase nuclei was based on the method of [21] and involved a detergent treatment (Fig. 2: step 3) with CHAPS (0.3%), Triton X-100 (0.3%), and Igepal (0.3%) added to PBS, followed by a short pepsin endopeptidase digestion (Fig. 2: step 4). Microscopic observation of counterstained nuclei has been instrumental in the timing of this step: cells with apparent cytoplasm and intact nuclear morphology can withstand a longer (up to 5 min) treatment and slightly deformed cytoplasm-free nuclei required a shorter, 2–3 min digestion.

The permeabilised nuclei were briefly post-fixed (3 min) in PFA-Igepal (Fig. 2: step 5a) and, after probe denaturation (Fig. 2: step 5b), the slides (now including the probe mixture) were denatured again (Fig. 2: step 6) at a high temperature (75 °C, 6 min). After testing several denaturation times (3–4–4.5–5–5.5–6 min), 6 min gave the most reliable signal-to-noise ratio (results not shown).

We also tested the effect of probe length delivered by different durations of the nick-translation labelling on the quality of immunoFISH-GISH in somatic nuclei of a wheat-barley translocation line (Additional file 1: Fig. S4). Tissue fixation was carried out for 1 h (5 + 55 min), 30 min (5 + 25 min) or 13 min (5 + 8 min) in 4% PFA-0.5% Igepal to also reveal the optimal fixation time for the immunoFISH-GISH procedure. The probe length (300–500 bp, determined by gel electrophoresis) was controlled by fragmenting the total DNA of barley prior the nick-translation labelling in a pressure cooker for 6 min. To visualise the barley chromosome segment in the translocation line, the fragmented total barley DNA was labelled by nick-translation for either 60 min (longest fragments), 100 min (shorter fragments), or 120 min (shortest fragments). Nuclei fixed for 1 h did not exhibit specific GISH signals when hybridised with any of the prepared probes (60 min-, 100 min- or 120 min-labelled). Similar results were obtained when hybridising the 30 min-fixed and 13 min-fixed nuclei with the longest, 60 min-labelled probes. Highly specific signals were detected when the probes had been labelled for 100 min (only for 5+8 min of fixation) or 120 min (Additional file 1: Fig. S**4**: framed panels). We thus concluded that fixations between 13–30 min and probes labelled for at least 100 min are needed to observe specific signals during immunoFISH-GISH.

### Applications of the optimised procedure

#### Immunolabelling of somatic and meiotic nuclei

**Fig. 3:**
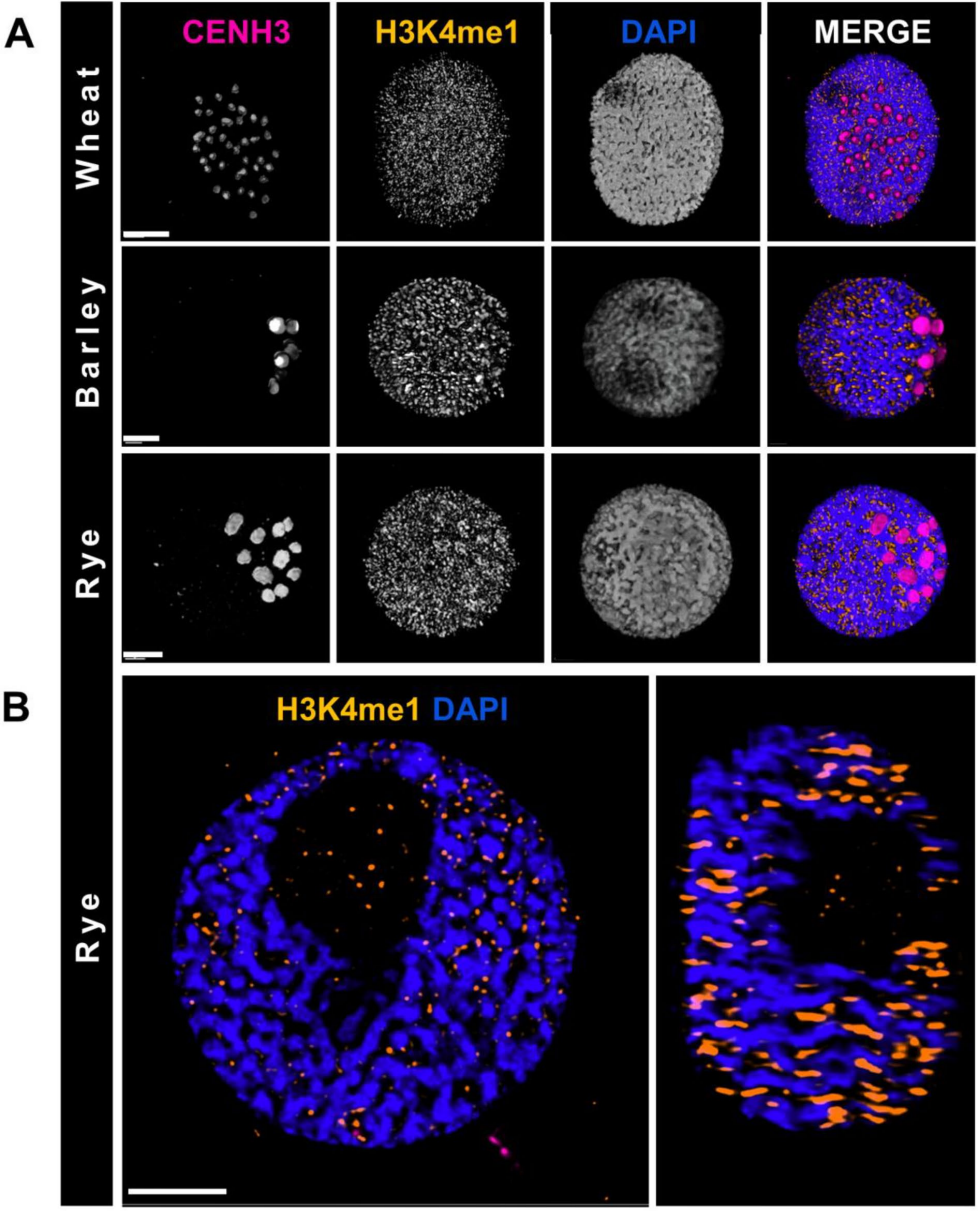
Immunolabelling of somatic nuclei prepared with the non-enzymatic in-solution procedure. **A** Single channel (monochrome) and merged images of 3D-rendered nuclei of wheat, barley and rye labelled by anti-centromeric histone H3 (anti-CENH3, magenta on merge) and anti-H3K4me1 (orange on merge) antibodies. The chromatin is counterstained with DAPI (blue on merge). **B** A single frame and side view of the immunolabelled (anti-H3K4me1, orange) rye nucleus presented in** A** (above). The chromatin is counterstained with DAPI (blue). Bars = 5 µm

We applied our procedure to prepare interphase/prophase nuclei from root tips of wheat, barley, and rye for immunolabelling with anti-CENH3 and anti-H3K4me1 antibodies (Fig. 3). The centromeric histone 3 (CENH3) labels indicate the position of active centromeres and the mono-methylated epigenetic modification at lysine 4 in histone H3 is associated with transcribed DNA sequences in plants [22] and thus marks gene-rich euchromatic regions [23, 24]. Due to its hexaploid state, the number of centromeric signals in the wheat genome is much higher than that in the diploid barley and rye (Fig. 3A). Immunolabelling indicated in all these species a polarised positioning of the centromeres while the histone epigenetic marks showed a relatively even distribution in blocks over the whole chromatin. This latter arrangement agrees with findings on the nuclear distribution of H3K4me1 in several plant species [23, 25, 26] and is consistent with data and the concept that euchromatic blocks are scattered over distal chromosomal regions in plants [25, 27–30].

**Fig. 4:**
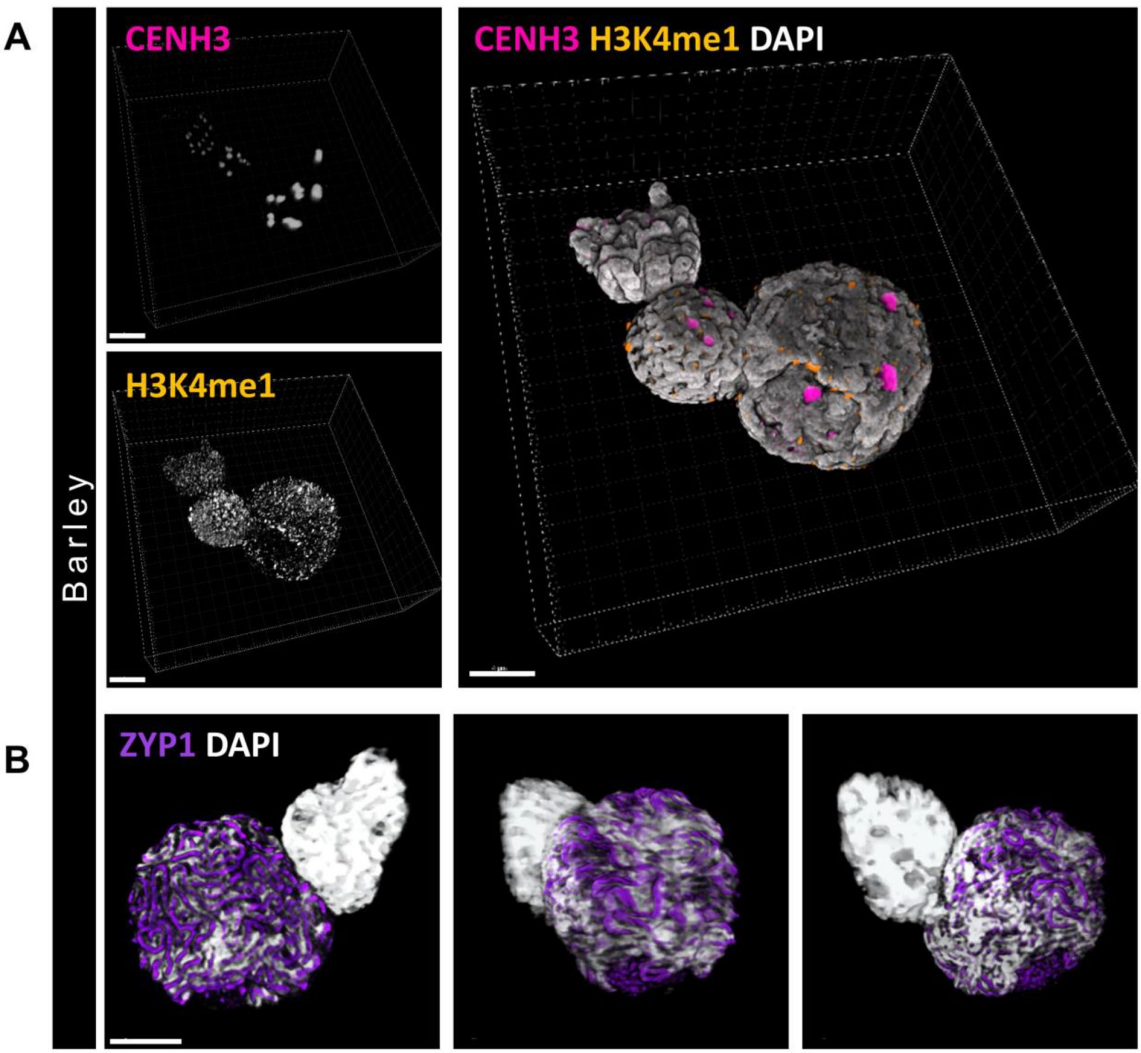
Immunolabelling of barley meiotic nuclei prepared with the non-enzymatic in-solution procedure. **A** Single channel (monochrome) and merged images of a 3D-rendered meiotic nucleus along with two mitotic nuclei. Active centromeres are labelled by an anti-CENH3 antibody (magenta on merge) and the mono-methylated H3 histone sites labelled by an anti-H3K4me1 antibody (orange on merge). The chromatin is counterstained with DAPI (white-grey). **B** Immunolabelling of the synaptonemal complex transverse filament protein (ZYP1, purple) on a meiotic nucleus next to a mitotic nucleus. The chromatin is counterstained with DAPI (white). The images show the 3D-rendered meiotic nucleus from three different angles. Bars = 5 µm

Prophase I. stage nuclei from male meiocytes of barley were immunolabelled (Fig. 4) as above demonstrating the reliability of the nuclei preparation procedure in different types of cells and tissues. Additional labelling with an antibody for the meiosis-specific synaptonemal complex transverse filament protein (ZYP1, Fig. 4B) was also compatible with the procedure.

#### ImmunoFISH

**Fig. 5:**
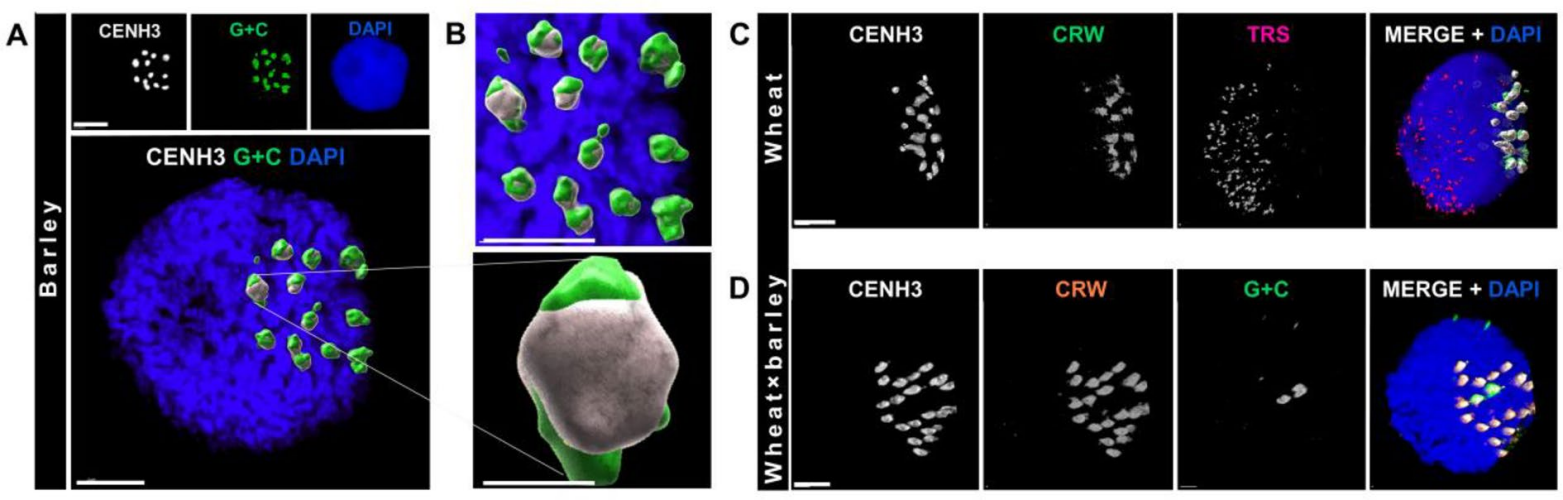
ImmunoFISH detection of somatic nuclei prepared with the non-enzymatic in-solution procedure. **A** Top row: single channel (monochrome) images of a 3D-rendered barley somatic nucleus. The CENH3 protein is shown in white (immunolabelling) and the barley centromere-specific G+C-rich satellite is shown in green (FISH). The chromatin is counterstained with DAPI (blue). Centromeric signals are shown in surface mode. The enlarged bottom image shows all channels merged into a single image. Bars = 5 µm. **B** Top image: an enlargement of the centromeres shown in the bottom of **A** (bar = 5 µm); bottom image: enlargement showing the structure of a single barley centromere (bar = 1 µm). **C** Single channel (monochrome) and merged immunoFISH images of a 3D-rendered wheat somatic nucleus. Active centromeres are labelled with an anti-CENH3 antibody (white on merge). The centromeric retrotransposon of wheat (CRW, green on merge) and the telomeric repeat sequences (TRS, magenta on merge) are detected by FISH. The chromatin is counterstained with DAPI (blue on merge). Bar = 5 µm. **D** Single channel (monochrome) and merged immunoFISH images of a 3D-rendered somatic nucleus of a wheat × barley F1 hybrid. Active centromeres are labelled with an anti-CENH3 antibody (white on merge). The centromeric retrotransposon of wheat (CRW, orange on merge) and the barley centromere-specific G+C-rich satellite (green on merge) are detected by FISH. The chromatin is counterstained with DAPI (blue on merge). Bar = 5 µm

The immunoFISH method was applied on barley, wheat and a wheat × barley F1 hybrid line to detect and colocalise the CENH3 protein with specific DNA sequences in 3D (Fig. 5). Optical sectioning showed the peripheral positioning of barley centromeric satellite repeats (G + C) and their partial colocalisation with the CENH3 protein, which demarcates the functional centromeres (Fig. 5A). High-resolution microscopy and 3D-rendering revealed, however, that the centromeric repeats expand beyond the CENH3-loaded centromeres indicating that not all these satellite repeats bind the CENH3 protein in barley (Fig. 5B). These observations are in agreement with the description of [31]. Similar results were collected in wheat, where the centromeric retrotransposons (CRWs) were detected by FISH parallel to labelling the active centromeres with a CENH3-specific antibody. Wheat CRWs partially colocalised but also stretched beyond the CENH3-rich core centromeres (Fig. 5C), indicating that the CENH3 protein is loaded only to a part of the CRWs in wheat somatic nuclei. The combined nuclei preparation and immunoFISH procedure also distinguished the barley and wheat centromeres in a wheat × barley F1 hybrid (Fig. 5D) as demonstrated by CENH3-labelling in combination with FISH using the G + C satellite and CRW probes, respectively. Two barley centromeres carried CENH3 and were thus active in the wheat background. Moreover, these barley centromeres colocalised with the wheat centromeres at the nuclear periphery (Fig. 5D) suggesting that they maintain the chromatin dynamics of the host genome.

#### ImmunoFISH-GISH

**Fig. 6:**
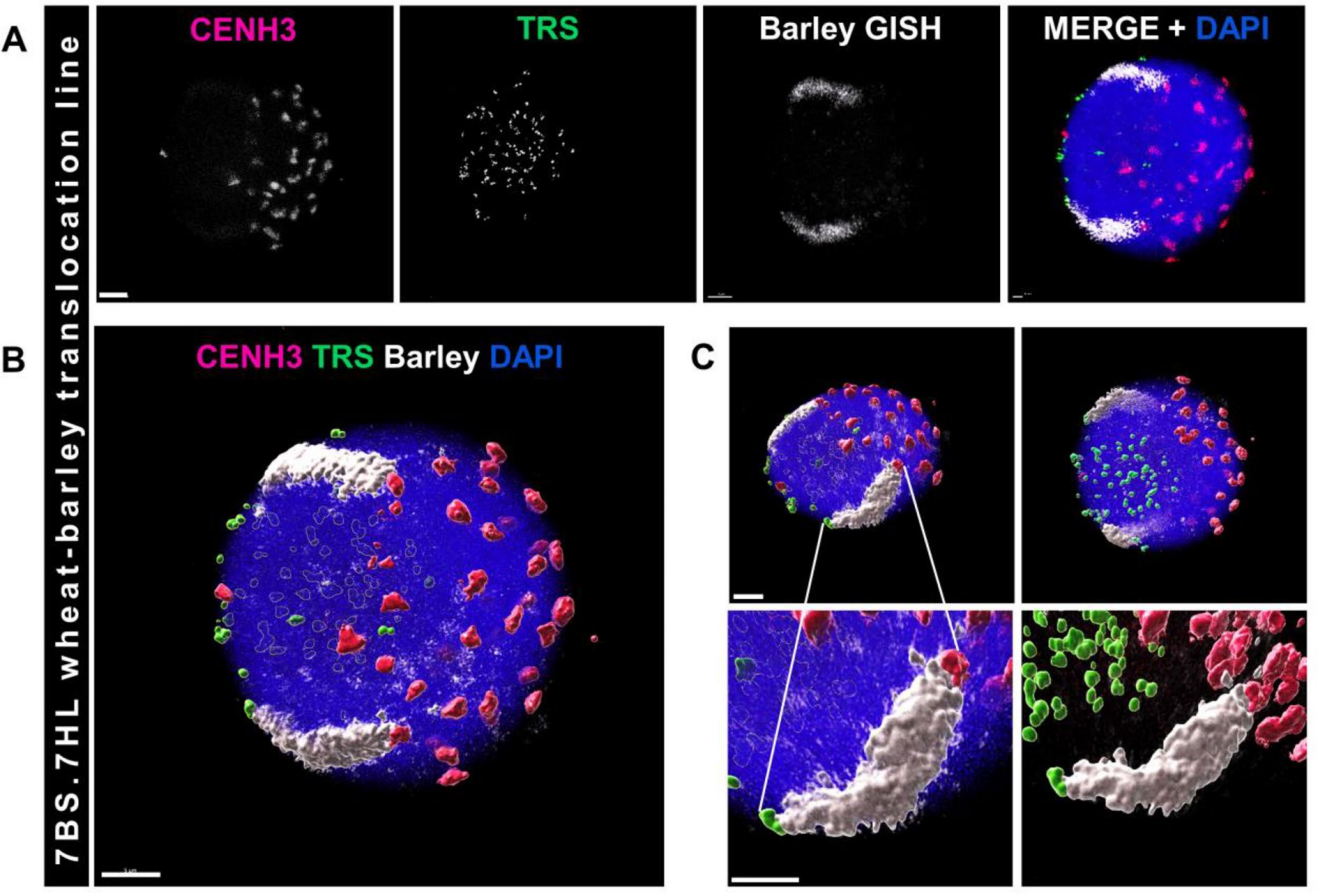
Combined immunoFISH-GISH detection of a somatic nucleus from a 7BS.7HL wheat-barley translocation line. **A** Single channel (monochrome) and merged images of a 3D-rendered nucleus. Active centromeres (CENH3 protein) are labelled with an anti-CENH3 antibody (magenta on merge), the telomeric repeat sequences (TRS) are visualised by FISH (green on merge), and the 7HL barley chromosome arm is detected by GISH (white on merge). The chromatin is counterstained with DAPI (blue on merge). **B** Surface rendering of the above nucleus labelled by immunoFISH-GISH. **C **Top row: the 3D-rendered nucleus on **B** is shown from two opposite sides. Bottom row: enlarged images of the 7HL barley chromosome arm (GISH, white), the active centromeres are immunolabelled (anti-CENH3, magenta), and telomeric repeat sequences (TRS) are detected by FISH (green). The chromatin is counterstained with DAPI (blue on merge). Bars = 5 μm

To be able to study the dynamics of alien chromatin in (cereal) hybrid plants and localise specific proteins inside the nucleus we applied the triple combination of immunolabelling, FISH and GISH (Fig. 6) on root tip nuclei, prepared by the described procedure, of a 7BS.7HL wheat-barley translocation line. The barley chromatin (a pair of the 7HL chromosome arm) was visualised by GISH in the context of telomeric repeats (TRS FISH) and active centromeres revealed by CENH3 immunolabelling (Fig. 6A, B). Barley centromere activity was confirmed by the presence of a distinct CENH3 signal on one end of the relevant chromosome arm, while the other end, located in the opposite nuclear hemisphere, was detected by the TRS probe (Fig. 6C). As a result, immunoFISH-GISH precisely outlined the physical as well as functional regions of the introgressed barley chromosome arm.

## Discussion

Several critical factors had to be optimised before the procedure could be applied consistently.

First, the typical PFA fixation times of several hours in traditional protocols (e.g., [32, 33]) and 1 h in our standard procedure [21] were reduced to 15–30 min so that the immunoreactivity of nuclear protein epitopes was better preserved. As a result of this short times of fixation, only covalent bonding of PFA is likely to occur instead of its usual cross-linking between proteins and nucleic acids [34, 35] that significantly compromises immunodetection. Formaldehyde is the oldest fixative [36], and, despite its toxicity and carcinogenicity, it is still one of the most popular fixatives and reported to outperform its proprietary alternatives [37].

Second, the addition of a detergent was used to enhance the penetration of PFA and to compensate for the shortened fixation time (see above). As an added benefit, this modification keeps the chromatin in a relaxed state [38, 39], thus counteracting its artificial condensation during subsequent steps (see below). Previously, the non-ionic and non-denaturing detergent Nonidet P-40 was occasionally added to fixatives [40]. Since Nonidet P-40 has been no more available, the equivalent Igepal CA-630 [41] was applied here.

Third, it was essential to replace enzymatic treatment with tissue grinding for cell wall breakdown. The non-denaturing PFA fixes proteins around the DNA fibres making them less accessible to labelled FISH/GISH probes. The interphase/prophase chromatin represents a further challenge because it is enclosed in the double membrane of the nuclear envelope as a barrier to the labelled probes. Mechanical disintegration of plant tissues for nuclei extraction has long been elaborated [42] and adapted to immunolabelling as well as in situ hybridization [43]. In our hands, the tissue grinder originally developed for soft tissues [44] delivered reproducible results when combined with subsequent filtering to remove cell debris. This debris is considered to cause non-specific antibody/probe binding and thus, as a source of background/noise signal, hampers the generation of high-resolution images.

Fourth and finally, for the simple in-solution approach a buffer supporting nuclear integrity had to be adopted. Out of three solutions commonly used for nuclei handling, the LB01 buffer appeared outstanding in this respect. The low aggregation of nuclei and reproducible signal intensities observed with LB01 can be attributed to its components (see the “Materials and methods” section). The monovalent cations Na^+^ and K^+^ are known to stabilize chromatin structure [45] by preventing its intense condensation [46]. The polyamine spermine can substitute divalent cations in stabilizing nuclear proteins, while the chelator EDTA inhibits phenol oxidases and nucleases by immobilizing their metal cofactors (Mg^2+^, Mn^2+^, and Cu^2+^). The inhibition of these enzymes is important in protecting nuclear proteins and the DNA from oxidation and denaturation. The non-ionic surfactant Triton X-100 aids the lysis and solubilisation of plastid membranes [47] and thus contributes to decreased cytoplasmic contamination.

The aim of this study was to develop a simple and versatile plant nuclei preparation procedure suitable for multiple purposes such as immunolabelling or in situ hybridisation alone but also the combination of these methods. We intended to include immunoFISH-GISH into the repertoire of molecular cytology to study chromatin dynamics and genome interaction in hybrid plants by detecting simultaneously selected loci (e.g., repetitive sequences, single-copy genes, and landmark chromosomal regions) and specific nuclear proteins.

The key factors in this procedure were improved fixation, the substitution of cell wall degrading enzymes with mechanical tissue grinding, and the introduction of in-solution preparation and pipetting of nuclei to microscope slides rather than manually releasing them from the corresponding tissues. To make up for the limitations caused by the reduction in fixation times we facilitated this step by vacuum infiltration and the inclusion of a non-ionic detergent.

Compared with the alternatives currently available the present procedure is simple, easily transferable, and feasible for non-experts in the field of cytology. The total duration of the procedure from fixation to preparation-ready cell nuclei is about half as much (ca. 45 min vs. 90 min) with a similar hands-on time as in our standard procedure [21]. The omission of the cell wall degrading enzymes (e.g., [48–51]) and of the manual preparation [52] makes the procedure more reliable and time efficient. The main time spare points compared to the listed protocols are the short duration of fixation (15–30 min vs. 1–2 days) and the elimination of enzymatic digestion (1–4 h).

We would like to emphasise, however, that the combination of immunolabelling and in situ hybridisation methods in plants are highly challenging due to the contrasting requirements for fixation and the difficulty imposed by the cell walls. The present nuclei preparation procedure and the immunoFISH-GISH method are closely linked to each other, therefore the combination of this preparation procedure with a different immunoFISH method (and *vice versa*) may lead to less satisfactory results.

At the dawn of modern plant breeding, especially of cereals, several successful, founder varieties turned out to carry chromosome segments integrated from related wild species [53]. The best-known examples are the interspecific 1R(1B) chromosome substitutions and the 1RS.1BL translocations introduced from cultivated rye into hexaploid wheat [54, 55]. While the translocation can be diagnosed by various biochemical and molecular methods [56], the initial molecular cytogenetic evidence for the 1RS.1BL translocation was provided by GISH [57]. Here, we used, for the first time, immunoFISH-GISH to analyse another Robertsonian translocation (7HS.7DL [58]) and demarcated the introgression by its functional boundaries, i.e., the active centromere via immunolabelling and the telomeric end via FISH. This is a strong case for the combination of fluorescence detection tools when the alien chromatin is in a well-defined nuclear context and the orientation of the chromosome arm can be revealed by labelling its landmarks. Another powerful application would be monitoring the differential interaction of the alien chromatin in various stages of meiosis and the process of recombination.

Analogously to hyphenated methods in metabolomics (e.g., LC-MS or GC-MS/MS), the described nuclei preparation procedure allows various combinations of immunolabelling and in situ hybridisation methods in plant cytogenetics. Two of these combinations, immunoFISH and immunoFISH-GISH, have been presented here and applied to somatic nuclei. Combining immunolabelling with 3D FISH and/or GISH creates a unique tool to assess the specific interactions and associations between proteins and nucleic acid sequences within the nucleus and this at the single cell level [13]. Thus, immunoFISH-GISH combined with the nuclei preparation procedure will facilitate more refined studies of chromatin dynamics, packaging, and stability throughout the cell cycle.

For the future, this procedure could be tested with various super-resolution microscopy platforms [59, 60] and adapted to alternative, PFA-free fixatives [61, 62]. Other challenges to solve will be the use of multiple probes for immunolabelling and/or FISH in interphase/prophase nuclei as well as in metaphase chromosomes. Integration with newly developed imaging workflows [63] would be an additional argument for the widespread application of the procedure.

## Conclusion

This study describes the development of a procedure for the preparation of high-quality 3D plant cell nuclei, which can reproducibly be applied to immunolabelling and in situ hybridisation methods as well as their combinations.

To the best of our knowledge, this is the first methodical description of a combined immunoFISH-GISH procedure in plants. The successful introduction of this triple-combined sophisticated methodology proves the high-quality of the plant nuclei prepared by the described procedure.

## Materials and methods

### Plant material

The materials used in the present study to obtain mitotic or meiotic preparations were the hexaploid wheat (*Triticum aestivum* L.) landrace ‘Chinese Spring’, the six-row spring barley (*Hordeum vulgare* L.) ‘Morex’ [64], the rye (*Secale cereale* L.) ‘Lovászpatonai’, a stable and fertile wheat-barley Robertsonian translocation line with the short arm of 7B wheat chromosome fused to the long arm of the 7H barley chromosome (7BS.7HL translocation, [53]), and a wheat × barley F1 hybrid (37/2020, 2*n* = 23, 21ABD + 2H chromosomes) from a cross between a Chinese spring wheat (‘Sichuan’)-derived doubled maternal haploid line, M1 (produced by previous pollinations with barley) and the ‘Golden Promise’ barley as described earlier [65].

### Collection of starting material

Seeds of wheat, barley, rye, and the 7BS.7HL wheat-barley translocation line were germinated in Petri dishes containing filter paper and an excess amount of water (50% distilled water-50% tap water). Following incubation at room temperature for 24 h, the excess water was removed, and the seeds were kept on wet filter paper for another 48 h to allow coleoptile emergence and root growth. When the root lengths reached 1–2 cm, root tips were collected and placed into the fixative.

For the wheat × barley F1 hybrid, roots were collected from potted plants placed for 1 week into trays containing ~ 1 cm water. The emerging white roots were transferred to 1× PBS (phosphate-buffered saline: 137 mM NaCl, 2.68 mM KCl, 10 mM Na_2_HPO_4_, and 1.76 mM KH_2_PO_4_, pH 7.4) and used for fixation.

Anthers were collected from the ears of ‘Morex’ barley as described [21]. Briefly, ears estimated to be entering meiosis were collected approximately 3 h after the light had come on. One of the three anthers per floret was squashed in 1% (w/v) acetocarmine stain and inspected under a phase contrast microscope to identify the approximate meiotic stage while the remaining anthers were placed into the fixative.

### Fixation

Root tips were immersed in ice-cold 4% PFA (freshly diluted in 1X PBS from isotonic 16% (w/v) Paraformaldehyde Solution; Thermo Scientific, Waltham, MA, USA, 28908) containing 0.5% (v/v) Igepal CA-630 (Sigma-Aldrich, St. Louis, MI, USA, I8896) and vacuum infiltrated for 5 min. After releasing the vacuum, the root tips were kept on ice and immersed in the fixative for another 8 or 25 min (see the Results section), then washed two times for 5 min in ice-cold PBS. Anthers were fixed as above for 5 min under vacuum, then for an additional 8 min, both on ice.

### In-solution preparation of nuclei

Fixed root tips or anthers cut with a razor blade were transferred into a 2 mL KIMBLE Dounce tissue grinder equipped with pestles A (large clearance) and B (small clearance) (Sigma-Aldrich, D8938; [44]) followed by adding 1 mL of LB01 buffer (15 mM Tris–HCl, 20 mM NaCl, 80 mM KCl, 0.5 mM spermine, 2 mM Na_2_EDTA, and 0.1% (v/v) Triton X-100, pH 9.0) [20]. The tissues were homogenised with pestle A for one min and for an additional four minutes with pestle B, which resulted in a homogeneous cell suspension, lacking an apparent debris. The suspension was then filtered through a 70-µm and 40-µm cell strainer (pluriStrainer Mini 70 µm, 43-10070-40 and pluriStrainer Mini 40 µm, 43-10040-40; pluriSelect Life Science, Leipzig, Germany). The filtered cell suspension was centrifuged at 2000 × *g* and 4 °C for 5 min. The supernatant was removed, and the pellet was resuspended in 50 µL of LB01 buffer. Five to eight µL of cell suspension were pipetted onto adhesion microscope slides (Epredia Superfrost® Plus Adhesion Microscope Slides; Menzel-Gläser, Braunschweig, Germany) and allowed to air dry (8–10 min). The specimens were either used directly for the cytological experiments or frozen on dry ice and stored for several weeks or months at – 80 °C.

### Immunolabelling

The immunolabelling and in situ hybridisation methods were based on our published procedure [21] with major modifications. The primary antibodies included a rabbit antibody raised against the CARTKHPAVRKTK peptide in the N-terminus of the wheat centromeric histone H3 protein (CENH3) [66, 67], a mouse anti-H3K4me1 antibody (Diagenode, Seraing, Belgium, C15200150), and, for meiotic cells, a rat anti-ZYP1 antibody [67, 68]. The ZYP1 antibody was prepared in a custom immunisation program against a recombinant protein covering a 424 AA sequence within the C-terminal region of the *Arabidopsis thaliana* ZYP1 protein as described [68].

The primary antibodies were diluted in 1X TNB blocking buffer (0.1 M Tris-HCl, pH 7.5, 0.15 M NaCl, and 0.5% (w/v) blocking reagent, Sigma-Aldrich, 11096176001) containing 0.3 M glycine (Sigma-Aldrich, G8898), 0.2% (v/v) Triton X-100, 0.2% (v/v) Igepal CA-630, and 0.025% (w/v) Saponin (Sigma-Aldrich, 47036) solution, at a ratio of 1:100 (CENH3) and 1:300 (H3K4me1). The slides were incubated with the primary antibodies at 37 °C for 1 h and transferred to 4 °C for overnight incubation. The next day the slides were incubated at 37 °C for 1 h before the primary antibodies were washed down two times for 5 min in PBS. The following secondary antibodies were applied depending on the respective primary antibody (see above): two goat anti-rabbit IgGs, Star Orange and Star Red (STORANGE-1002 and STRED-1002, Abberior GmbH, Göttingen, Germany), a goat anti-mouse IgG, Star Green (STGREEN-1001, Abberior), and a goat anti-rat IgG, Star Green (STGREEN-1007, Abberior). The secondary antibodies were diluted to 1:150 in 1× TNB blocking buffer, then incubated at 37 °C for 45 min. The slides were then washed (two times 5 min in PBS) and either immediately processed for in situ hybridisation or mounted (Vectashield Antifade Mounting Medium with DAPI; Vector Laboratories, Newark, CA, USA, H-1200).

### In situ hybridisation

#### Probe preparation and labelling

To prepare the FISH probes, the barley centromere-specific G + C-rich satellite sequence [69], part of the wheat centromeric retrotransposon (CRW [66]), and the universal plant telomeric repeat sequence (TRS [70]) were amplified by PCR. The GISH probe was prepared from total DNA extracted with a standard CTAB method from fresh young leaves of ‘Manas’ barley.

Amplified DNA sequences (FISH) or total barley DNA (GISH) were directly labelled by nick-translation (AF488 NT Labeling Kit, PP-305L-AF488; AF594 NT Labeling Kit, PP-305L-AF594; and AF647 NT Labeling Kit, PP-305L-AF647; Jena Bioscience, Jena, Germany). FISH probes were labelled in a PCR machine at 15 °C for 60 min. For the GISH probes, heat-fragmented (6 min, pressure cooker) total DNA was labelled at 15 °C for 100 min.

#### Pretreatments

Cell permeabilisation was ensured by pipetting 50 µL of 1× PBS containing 0.3% (w/v) CHAPS zwitterionic surfactant (Sigma-Aldrich, C3023), 0.3% (v/v) Triton X-100, and 0.3% (v/v) Igepal CA-630 on the slides and covering them with a plastic coverslip. The slides were incubated in a moisture chamber at 37 °C for 15 min and washed in 1× PBS at room temperature for 5 min followed by a quick, 10 s rinse.

Further membrane permeabilisation was achieved by incubating the slides with 50 µL of prewarmed pepsin solution (50 µg/mL, Sigma-Aldrich, P6887) under a plastic coverslip at 37 °C for 5 min. After washing two times in 1× PBS for 5 min, 50 µL of 4% PFA-Igepal CA-630 solution (see Fixation above) was added to the slides and incubated at room temperature for 3 min under a plastic coverslip.

#### Fluorescence in situ hybridisation

The hybridisation mix contained 60% (v/v) deionised formamide (Sigma-Aldrich, F9037), 10% (w/v) dextran sulphate (Sigma-Aldrich, 67578) in 2× SSC (saline-sodium citrate). A volume of 17 µL per slide was completed with 40–60 ng of the labelled probe and denatured at 85 °C for 8.5 min in a PCR machine. The denatured probe mixture was immediately transferred to ice for at least 5 min. The ice-cold mixture was pipetted on the surface of permeabilised and post-fixed specimens and covered with a 22 × 32 glass coverslip. The slides were denatured at 75 °C for 6 min in a PCR machine equipped with a stainless-steel plate, then incubated at 37 °C for 5–18 h in a moisture chamber to allow hybridisation. Post-hybridisation washes were performed two times in 1× PBS at 37 °C for 5 min and the slides were mounted in 12 µL of Vectashield Antifade Mounting Medium with DAPI.

#### Simultaneous FISH-GISH

Fifty ng of labelled GISH probe per slide was pipetted together with 40-60 ng of FISH probe and an excess (30×) of CTAB-purified, unlabelled total wheat DNA (blocking) in 18 µL hybridisation mix per slide (see above, FISH procedure). Probe and slide denaturation, hybridisation, washes and mounting were carried out as described for FISH.

### Confocal laser-scanning microscopy

Detection of fluorescence signals was performed by an SP8 Tunable Confocal System (TCS: Leica Microsystems, Wetzlar, Germany). The DAPI-stained chromatin was detected between 410 and 470 nm after excitation at 405 nm. The settings for the applied fluorescent dyes conjugated to various secondary antibodies were as follows: Star Green and Alexa Fluor 488—excited at 488 nm, detected from 490 to 560 nm; Star Orange and Alexa Fluor 594—excited at 561 nm, detected from 600 to 660 nm; Star Red and Alexa Fluor 647—excited at 633 nm, detected from 650 to 700 nm. A series of confocal images (‘*z*-stacks’) with a lateral (*x* and *y*) resolution of 45 nm and an axial (*z*) resolution of 200 nm were acquired by an HC PL APO CS2 63×/1.40 oil immersion objective (Leica). The size of the confocal aperture was set to 1.35 Airy Units (128.9 µm). Image acquisition was carried out by bidirectional scanning along the *x*-axis and the images were averaged from three distinct frames to reduce image noise. *Z*-stacks were subjected to deconvolution by Huygens Essential v18.04 (Scientific Volume Imaging, Hilversum, the Netherlands) and 3D reconstructions were obtained using the Leica Application Suite Advanced Fluorescence software v3.1.5.1638 or the Imaris multidimensional microscopy data analysis software v9.6 (Oxford Instruments, Abingdon, the United Kingdom).

### Supplementary Information

**Additional file 1: Fig. S1.** The effect of a non-ionic detergent (Igepal) in the non-degenerating fixative (4% PFA) during the short fixation of wheat (top panels) and barley (bottom panels) root tips.

**Additional file 2:** **Fig. S2. **The effect three fixation times on the cell integrity (DAPI: top row) and permeability (immunoFISH: bottom row) in somatic nuclei of wheat (**A**) and (**B**).

**Additional file 3:** **Fig. S3. **The effect of three buffers used during tissue grinding on the in-solution aggregation of barley root tip cell nuclei.

**Additional file 4:** **Fig. S4.** The combined effect of three fixation times (panels) and probe lengths (rows) controlled by the duration of nick translation during probe labelling on the permeability of barley nuclei in the immunoFISH-GISH procedure.

## Data Availability

Not applicable: all data generated or analysed during this study are included in the paper.
