# Supplementary figures and images for: Rapid in-solution preparation of somatic and meiotic plant cell nuclei for high-quality 3D immunoFISH and immunoFISH-GISH
Source: Plant Methods. 2023 Aug 8;19:80. doi: 10.1186/s13007-023-01061-7 (PMC10408160; doi:10.1186/s13007-023-01061-7)

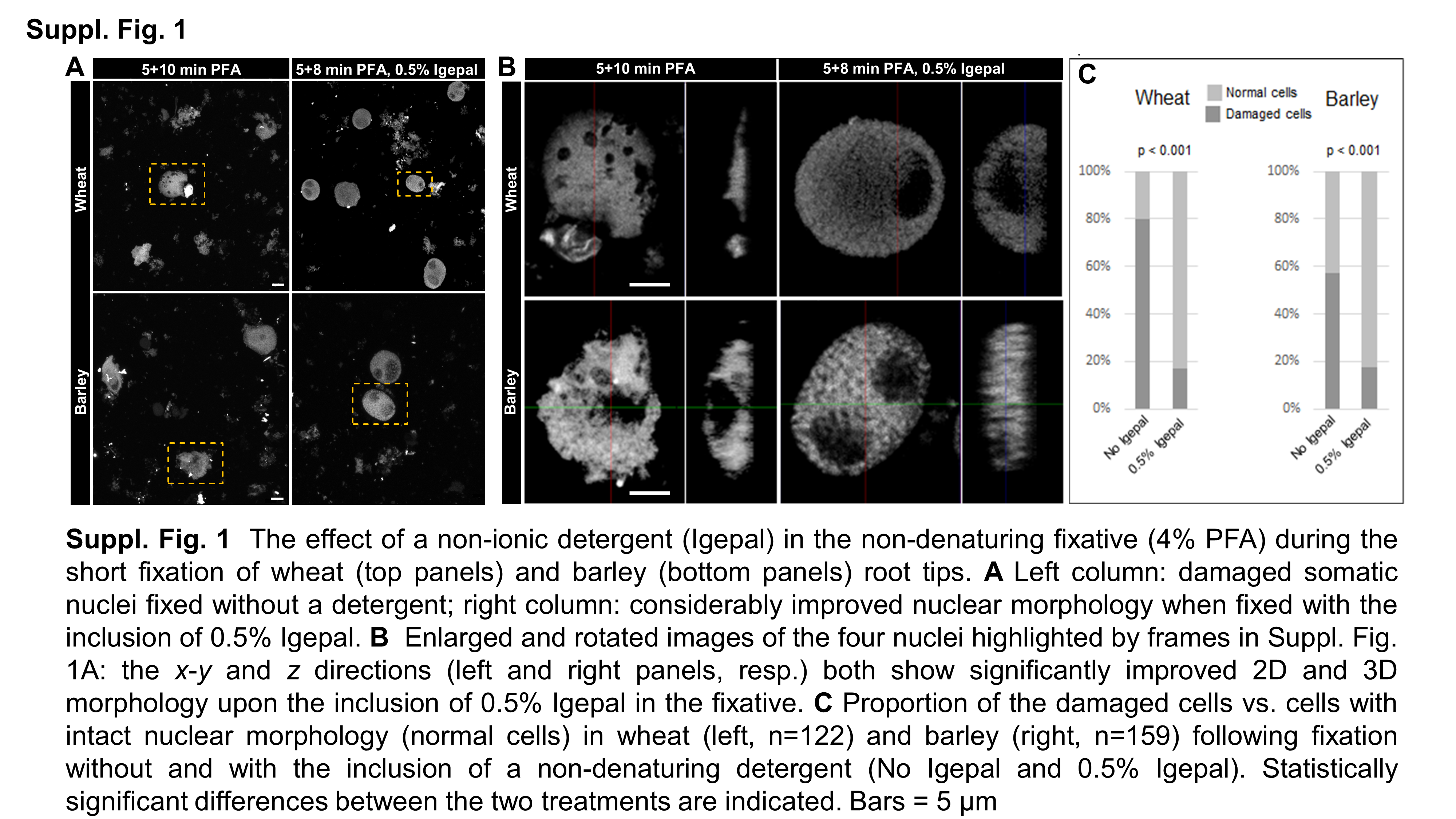

Supplement: Supplementary file 1 — Additional file 1: Fig. S1. The effect of a non-ionic detergent (Igepal) in the non-degenerating fixative (4% PFA) during the short fixation of wheat (top panels) and barley (bottom panels) root tips. [file 13007_2023_1061_MOESM1_ESM.tif]

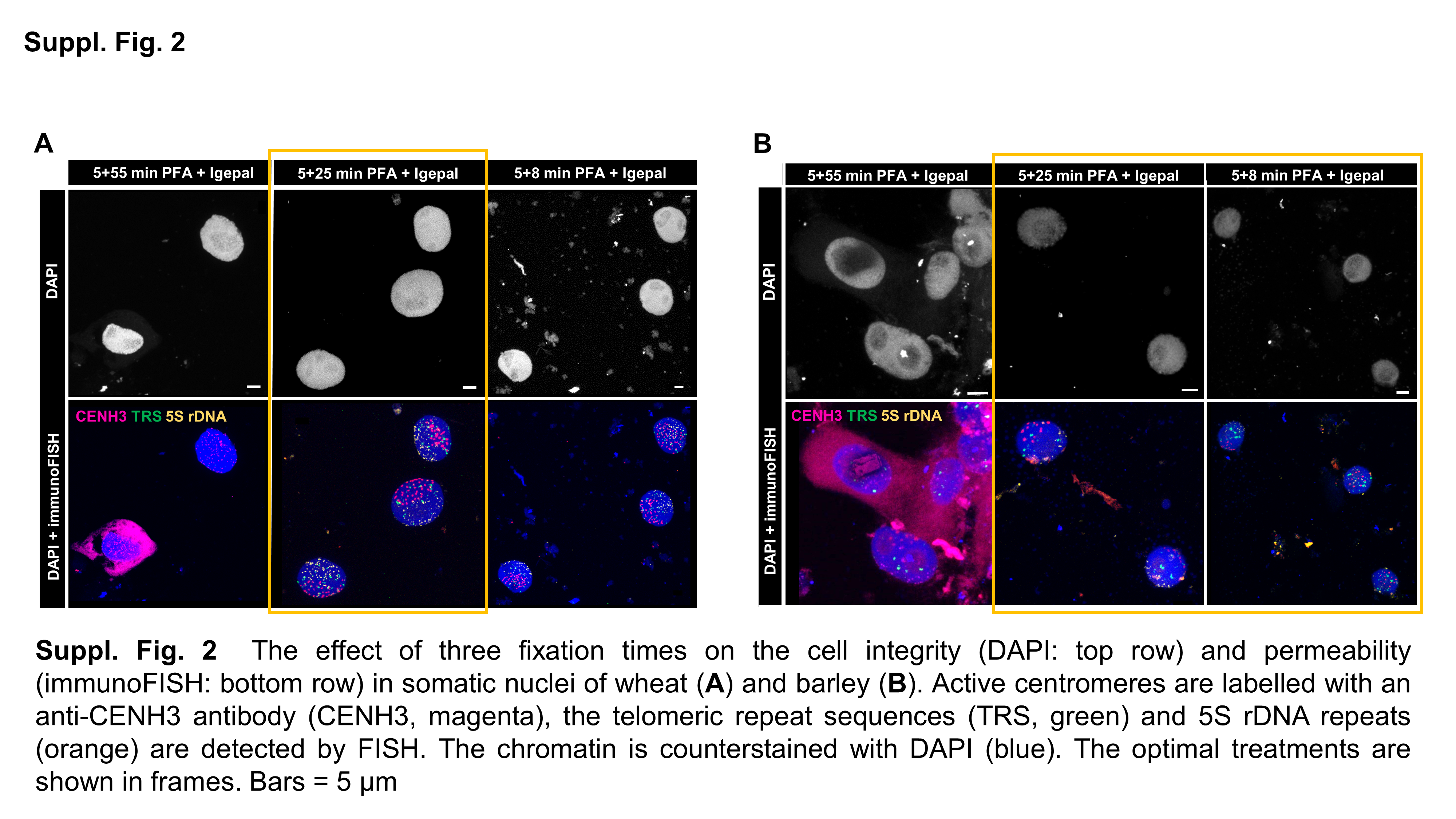

Supplement: Supplementary file 2 — Additional file 2: Fig. S2. The effect three fixation times on the cell integrity (DAPI: top row) and permeability (immunoFISH: bottom row) in somatic nuclei of wheat (A) and (B). [file 13007_2023_1061_MOESM2_ESM.tif]

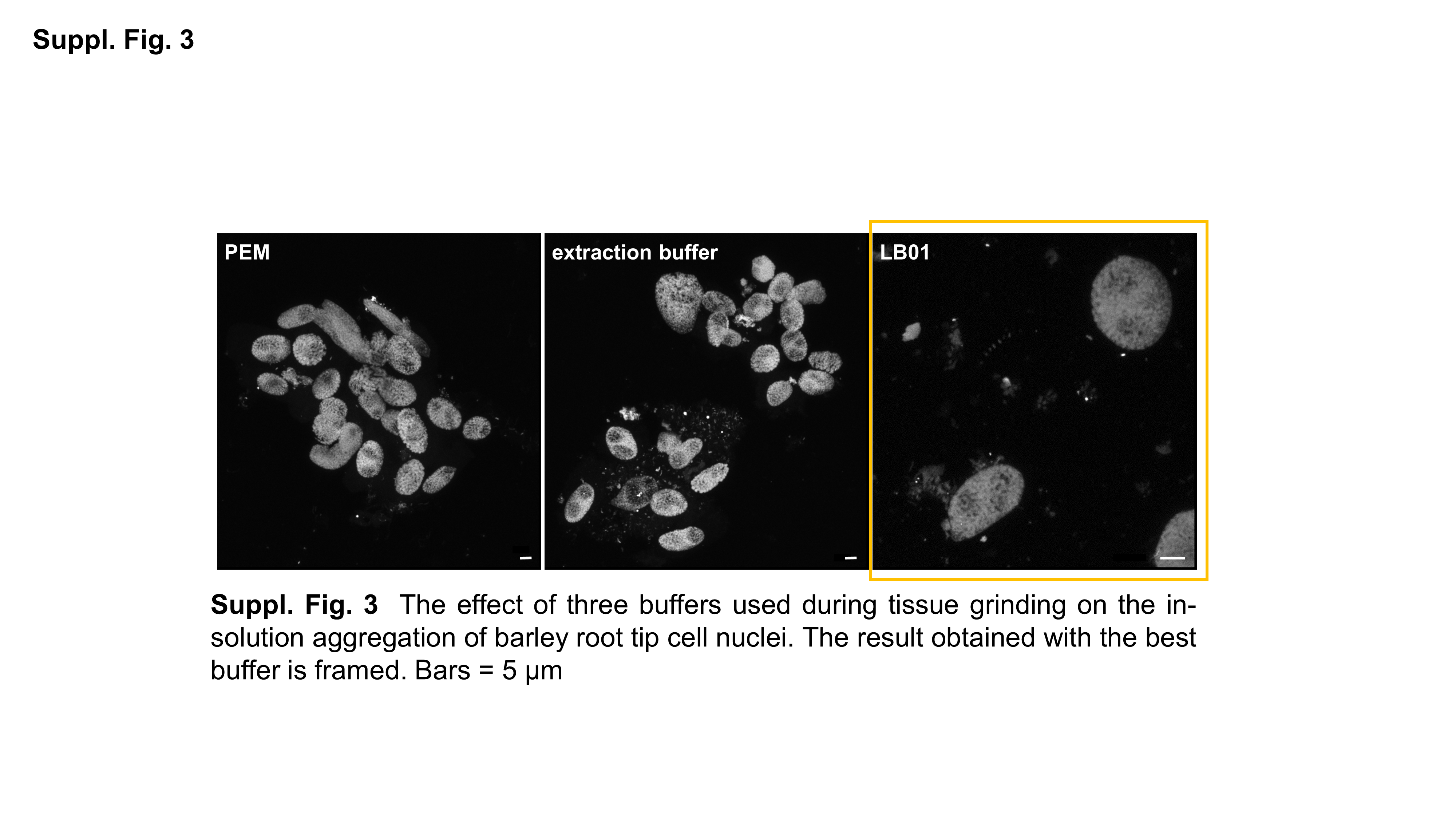

Supplement: Supplementary file 3 — Additional file 3: Fig. S3. The effect of three buffers used during tissue grinding on the in-solution aggregation of barley root tip cell nuclei. [file 13007_2023_1061_MOESM3_ESM.tif]

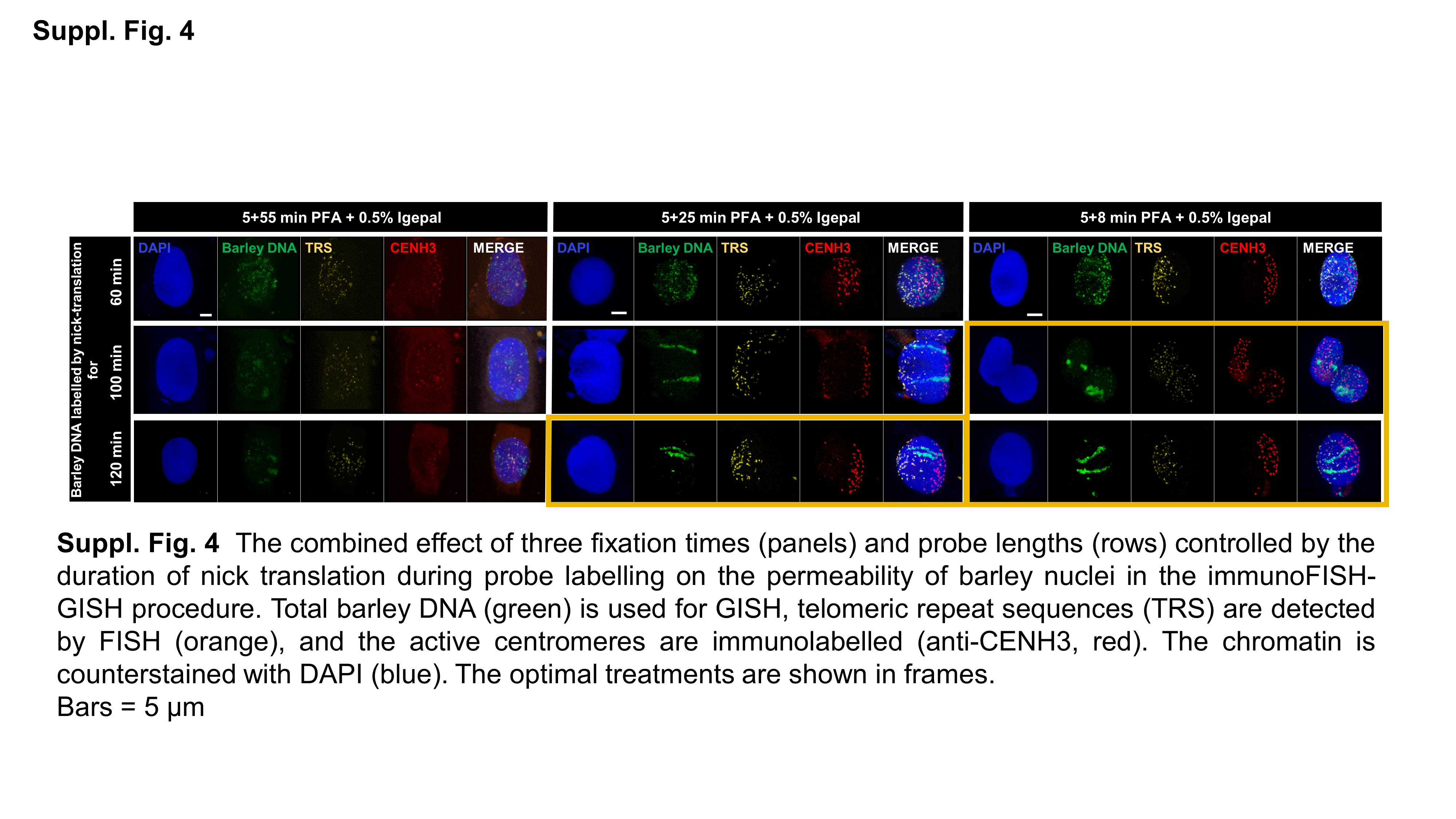

Supplement: Supplementary file 4 — Additional file 4: Fig. S4. The combined effect of three fixation times (panels) and probe lengths (rows) controlled by the duration of nick translation during probe labelling on the permeability of barley nuclei in the immunoFISH-GISH procedure. [file 13007_2023_1061_MOESM4_ESM.tif]
